# Louisiana State of Mind: Women’s Mental Health after the *Deepwater Horizon* Oil Spill

**DOI:** 10.1289/ehp.124-A170

**Published:** 2016-09-01

**Authors:** Nancy Averett

**Affiliations:** Nancy Averett writes about science and the environment from Cincinnati, OH. Her work has been published in *Pacific Standard*, *Audubon*, *Discover*, *E/The Environmental Magazine*, and a variety of other publications.

Studies of several different oil spill disasters have shown that the people affected by these disasters can suffer adverse mental health and behavioral effects.^1^ ^,^ ^2^ But does the psychological impact of this kind of event differ among subgroups of the affected population? A new study explores that question by looking at how the *Deepwater Horizon* oil spill in 2010 specifically impacted women’s mental health and likelihood of domestic conflict.^3^

“We felt women exposed to the spill were an understudied group [in terms of the *Deepwater Horizon* event],” says first author Ariane L. Rung, an associate professor at the Louisiana State University Health Sciences Center in New Orleans. Women are nearly 2.5 times more likely than men to experience abuse by an intimate partner.^4^ Yet women are also influential within the family structure. “They often make the day-to-day decisions that are central to families regarding financial matters, food preparation, and child care,” she explains.

**Figure:**
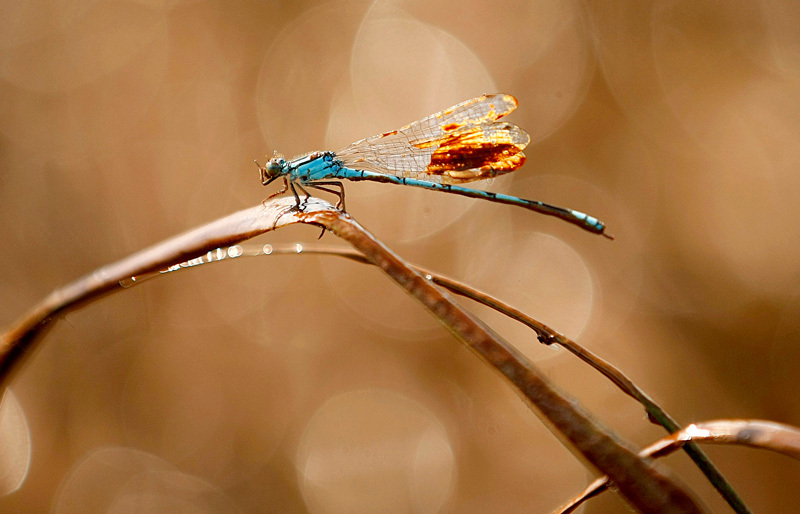
More than a quarter of the women in the WaTCH study reported symptoms of depression after the *Deepwater Horizon* oil spill, while 16% reported an increase in domestic conflict. © Associated Press

Rung and her coauthors used data from the first wave of interviews conducted for the longitudinal Women and Their Children’s Health (WaTCH) study. This study was designed to assess health effects of the *Deepwater Horizon* spill in nearby residents. The interviews involved 2,842 women in 7 southern Louisiana coastal parishes. Most of the women had graduated high school but not college, were non-Hispanic white, and were married or living with a partner.^3^

The researchers asked the women questions about what they termed economic and physical exposures to the oil spill. Economic exposures included whether participants had lost income due to the oil spill, whether the oil spill had a negative effect on their household finances, and whether they felt they had been hit harder by the spill than others in their community. Physical exposures included scenarios such as whether participants could smell the oil, whether they had come into physical contact with the oil in other ways, and whether the spill had directly affected their recreational activities such as hunting or fishing.

The women also completed standard scales to assess symptoms of depression and mental distress. Finally, they answered questions about the frequency and intensity of conflicts at home.

More than a quarter of the women reported having depressive symptoms, with 13% reporting symptoms that indicated severe mental distress. In addition, 16% said they had had more fights with their partners since the spill, while 11% said the intensity of their fights had increased.^3^

Rung and her coauthors note that they did not have data on the women’s mental health status prior to the spill. It is possible the women already had higher rates of depression before the spill occurred, perhaps related to disasters such as Hurricanes Katrina and Rita. The authors also acknowledge that there is no objective biomarker for measuring a person’s exposure to an oil spill. “We had to rely on people self-reporting what their exposure was,” Rung says.

Lawrence Palinkas, a professor of social policy and health at the University of Southern California, points out that although this particular investigation was cross-sectional, the WaTCH study itself is longitudinal. “It would not surprise me in the least if [follow-up studies] show symptoms are diminishing over time but that there are still long-term effects,” Palinkas says. He adds that there may also be subgroups of women who are more susceptible to these effects than others; for example, lower-income women may be more susceptible to prolonged effects. Palinkas was not involved in the study.

The lack of baseline mental health data is not uncommon in disaster studies. According to Palinkas, one way to validate the findings is to compare the outcomes to those of similar studies, such as work he conducted on the psychological aftermath of the 1989 *Exxon Valdez* oil spill in Alaska.^2^ He says Rung and colleagues found rates of self-reported adverse mental health markers that were similar to what he found among people who lived near the *Exxon Valdez* spill.

The researchers acknowledge that their questions pertaining to domestic violence were general in nature and not comprehensive descriptors of partner violence.^3^ “Positive responses to those kinds of questions could lead to questions about much more serious kinds of abuse,” Rung says. “But our study wasn’t designed to pick that up.”
